# Secondary Brain Injury Following Neonatal Intraventricular Hemorrhage: The Role of the Ciliated Ependyma

**DOI:** 10.3389/fped.2022.887606

**Published:** 2022-06-30

**Authors:** William Dawes

**Affiliations:** ^1^Alder Hey Children's Hospital, Liverpool, United Kingdom; ^2^NIHR Great Ormond Street Hospital BRC, London, United Kingdom

**Keywords:** intraventricular hemorrhage, post haemorrhagic hydrocephalus, ependymal cilia, cerebrospinal fluid, subventricular zone (SVZ)

## Abstract

Intraventricular hemorrhage is recognized as a leading cause of hydrocephalus in the developed world and a key determinant of neurodevelopmental outcome following premature birth. Even in the absence of haemorrhagic infarction or posthaemorrhagic hydrocephalus, there is increasing evidence of neuropsychiatric and neurodevelopmental sequelae. The pathophysiology underlying this injury is thought to be due to a primary destructive and secondary developmental insult, but the exact mechanisms remain elusive and this has resulted in a paucity of therapeutic interventions. The presence of blood within the cerebrospinal fluid results in the loss of the delicate neurohumoral gradient within the developing brain, adversely impacting on the tightly regulated temporal and spatial control of cell proliferation and migration of the neural stem progenitor cells within the subventricular zone. In addition, haemolysis of the erythrocytes, associated with the release of clotting factors and leucocytes into the cerebrospinal (CSF), results in a toxic and inflammatory CSF microenvironment which is harmful to the periventricular tissues, resulting in damage and denudation of the multiciliated ependymal cells which line the choroid plexus and ventricular system. The ependyma plays a critical role in the developing brain and beyond, acting as both a protector and gatekeeper to the underlying parenchyma, controlling influx and efflux across the CSF to brain interstitial fluid interface. In this review I explore the hypothesis that damage and denudation of the ependymal layer at this critical juncture in the developing brain, seen following IVH, may adversely impact on the brain microenvironment, exposing the underlying periventricular tissues to toxic and inflammatory CSF, further exacerbating disordered activity within the subventricular zone (SVZ). By understanding the impact that intraventricular hemorrhage has on the microenvironment within the CSF, and the consequences that this has on the multiciliated ependymal cells which line the neuraxis, we can begin to develop and test novel therapeutic interventions to mitigate damage and reduce the associated morbidity.

## Introduction

### Intraventricular Hemorrhage Is Common, Devastating and Without Treatment

In the UK around 60,000 babies are born prematurely each year with a degree of hemorrhage seen in around one third, equating to around 20,000 cases of neonatal intraventricular hemorrhage per year. The high metabolic demand of the developing brain, in association with fragile and underdeveloped cerebral vasculature and parenchyma, combine to make the premature neonate prone to hemorrhage within the germinal matrix (GMH) (1, 2); its incidence being inversely proportional to the degree of brain maturation (3).

Despite advances in neonatal care, premature birth remains common with up to 15 million babies born preterm (<37 weeks gestation) every year worldwide. Indeed, over the last two decades, studies indicate that rather than decreasing, the incidence of premature birth has actually increased in almost all countries with reliable data (4, 5). The reason for the observed increase is incompletely understood but is thought to be related to delayed primigravida and the increased use of fertility treatments in the developed world (6, 7).

**Figure 1:**
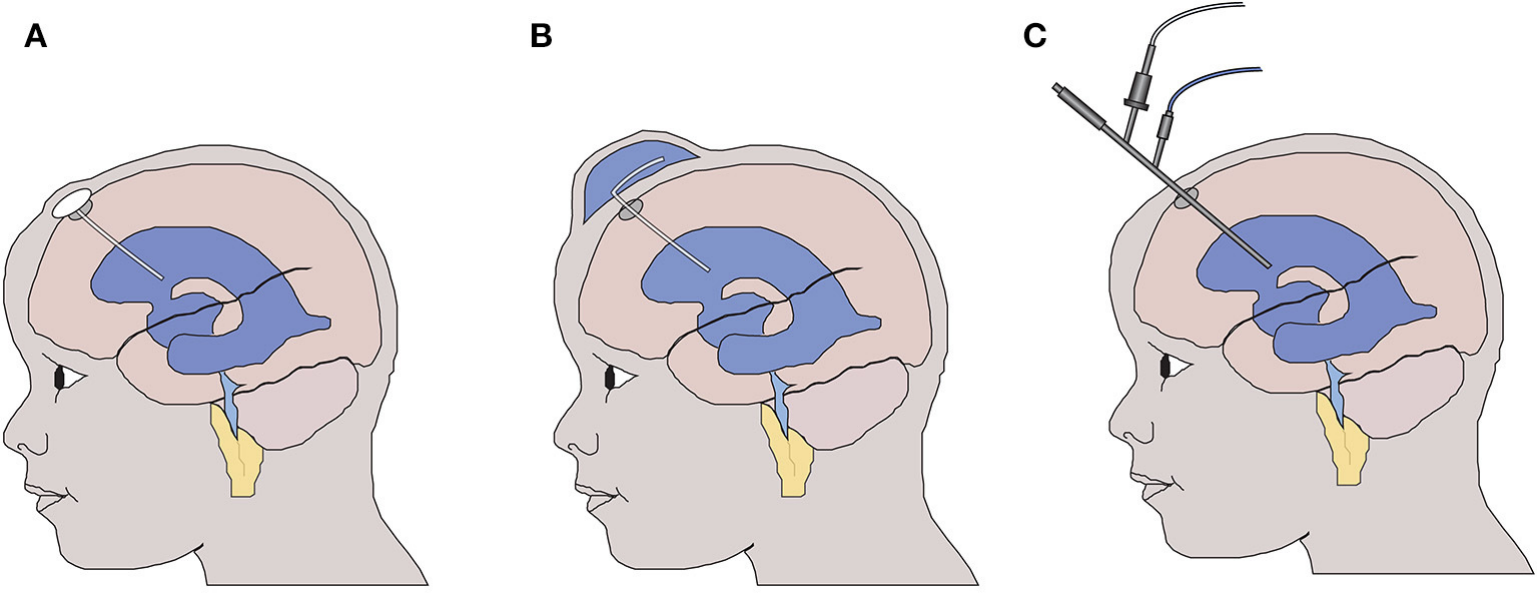
Temporizing interventions used for the treatment of neonatal post haemorrhagic hydrocephalus. **(A)** Ventricular access device – a CSF reservoir is inserted allowing intermittent aspiration of CSF to control intraventricular pressure. **(B)** Ventricular subgaleal shunt – the potential space between the galea and the periosteal lining of the cranium is surgically opened allowing CSF to drain into the subgaleal space. **(C)** Neuroendoscopic lavage of the ventricular system with or without septostomy to access the contralateral ventricle. The blood-stained CSF is removed to reduce the potential toxicity to the wall of the lateral ventricle.

In the majority of cases, neonatal intraventricular hemorrhage occurs within the first 4–5 days of life. This can lead to life threatening cardiovascular instability, indeed neonatal intraventricular hemorrhage (NIVH) remains a leading cause for the withdrawal of care. Following the identification of hemorrhage, early management priorities are supportive with no active therapy instigated. Twice weekly transfontanelle ultrasound scanning is used to monitor the anatomy of the ventricular system, and in those neonates who develop radiological signs (8) or symptoms of hydrocephalus, a temporizing intervention, aimed at reducing intraventricular pressure, is instigated at around 20–30 days of age (Figure 1). As such the current gold standard treatment, aimed at reducing intraventricular pressure with surgical intervention, does not address the immediate and continuing toxic impact of blood within the cerebrospinal fluid (9).

Incremental and focused research efforts over the past four decades have significantly reduced the incidence of severe NIVH (10), however the advances seen in prevention of NIVH, have not to date been matched by advances in treatment for those neonates who still suffer hemorrhage. Whilst magnesium sulfate has been shown in two metanalysis to reduce the risk of cerebral palsy following IVH (11, 12) unfortunately interventions such as the use of acetazolamide and frusemide (13–15) or early prophylactic lumbar punctures (16) have not been shown to improve outcome. Indeed the increased incidence of prematurity, in combination with the improving survival of smaller neonates, has led to a significant increase in the absolute number of infants (17, 18) with neurodisability after premature birth (19, 20). This has led to a real need to understand the mechanism of injury associated with intraventricular hemorrhage and to develop novel therapies to prevent its deleterious consequences. The most promising intervention, involving drainage, irrigation and fibrinolysis of the CSF (DRIFT) is discussed in more detail below (21, 22).

Within this review I will discuss the clinical and experimental evidence implicating that NIVH elicits a secondary injury process to the developing brain, specifically focusing on the role that damage to the ependymal lining of the ventricle may play in this process. The aim of this review paper is to stimulate interest in promoting research into maintaining ependymal health as a potential therapeutic avenue following neonatal intraventricular hemorrhage.

## Clinical Correlates of Neonatal IVH/Evidence of Secondary Injury Process

**Figure 2:**
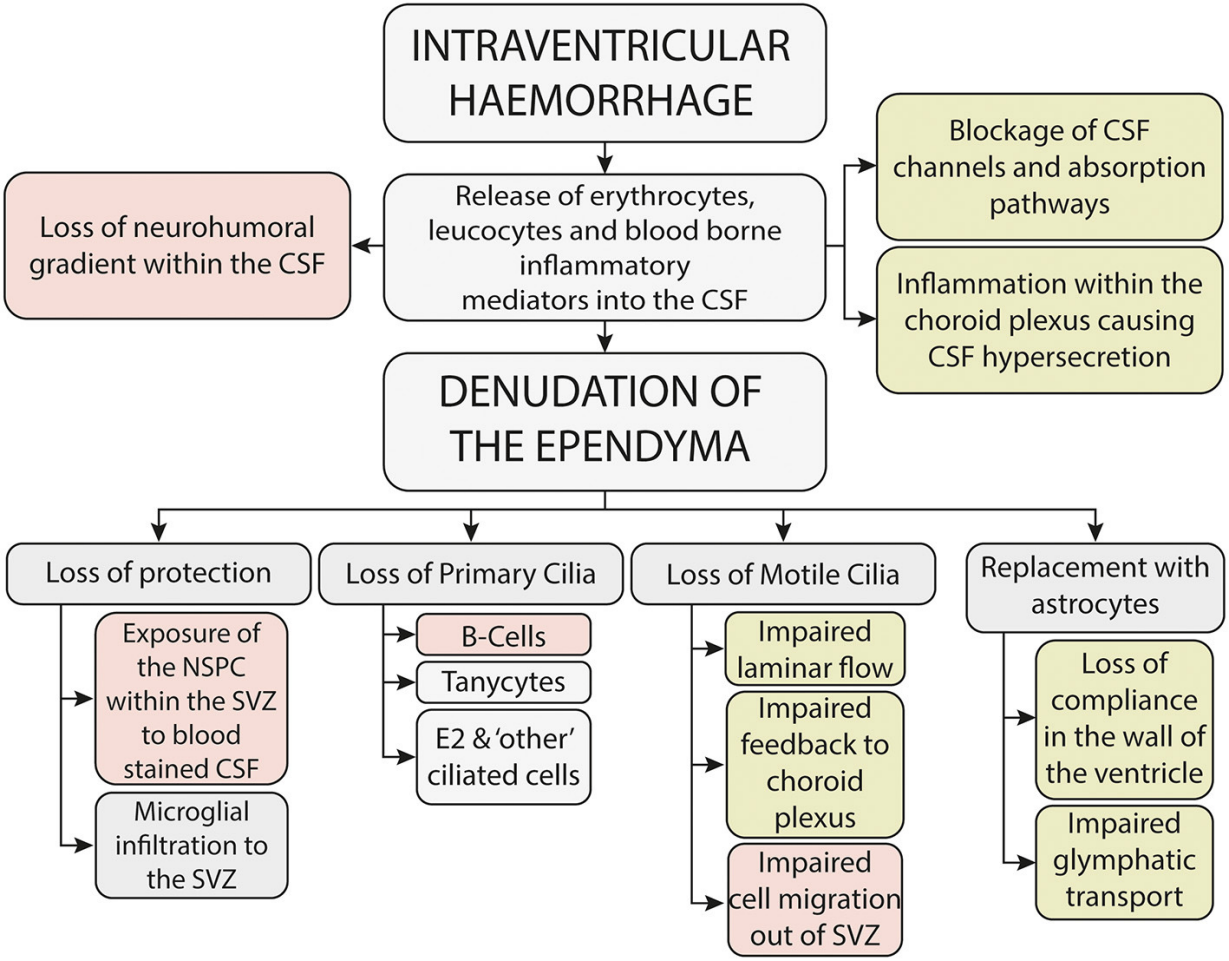
Potential mechanisms underlying the secondary brain injury seen following neonatal intraventricular hemorrhage-mechanisms potentially associated with neurodevelopmental outcome are shown in red, whilst mechanisms potentially associated with the development of hydrocephalus are shown in green.

Since Volpe's seminal paper in 2009 (23) it is widely recognized that IVH causes both an immediate-primary destructive injury, due in part to the kinetic energy released at the time of the hemorrhage, and a delayed, secondary-developmental injury to the brain parenchyma, which is thought to be mediated through the toxicity associated with blood within the parenchyma and cerebrospinal fluid (CSF) (9) (Figure 2). Support for a secondary injury process comes from multiple lines of enquiry, including the finding of global developmental and neuropsychiatric problems in neonates with mild IVH (24), the radiological finding of abnormalities in brain development, away from the site of injury and also from clinical evidence which implicates that washing away blood-stained CSF (22, 25) and intervening before the onset of clinical symptoms (26) may improve outcome.

### The Impact of IVH on Neurodevelopmental Outcome

Despite improvements in perinatal care, neonatal intraventricular hemorrhage (NIVH) remains a leading cause of neurodevelopmental delay in childhood and the commonest cause of hydrocephalus in the developed world (27).

Outcome studies following premature birth consistently show that a key determinant is the presence or absence of intraventricular hemorrhage (28). With high grade hemorrhage [classically defined using the Papile grading system (29)] associated with the worst outcomes. The impact of lower grades of hemorrhage remains contentious with conflicting reports published (30–33), but recent evidence implicates that even low grade neonatal intraventricular hemorrhage is associated with significant psychiatric sequelae. For example, an increased incidence of ADHD and tic disorders at 6 years of age as well as an increased risk of major depressive disorder and obsessive–compulsive disorder at 16 years of age has been shown (24).

The variability seen in neurodevelopmental outcomes is multifactorial with comorbidity and parental socioeconomic status (34) playing key roles. It is recognized however that the development of hydrocephalus necessitating ventricular peritoneal shunting (VPS) is a key predictor of outcome (35–37) and in this regard every effort has been made to reduce the rate of permanent VPS (15, 16, 21, 38). It is important to note however that whilst posthaemorrhagic hydrocephalus is likely to be a contributory factor to outcome, this is unlikely to be solely responsible for the observed neurodevelopmental delay. For example, the outcome following isolated neonatal hydrocephalus i.e., not related to IVH, is significantly better than that caused by IVH (39). Implicating that whilst important, hydrocephalus may not be the driver of outcome. It is also interesting to note that the burden of care in patients with hydrocephalus secondary to IVH is more significant than other causes of hydrocephalus (39).

### The Impact of IVH on Brain Volumes and Connectivity

With the advent of modern magnetic resonance imaging (MRI) modalities has come the ability to analyse the developing brain in previously unobtainable detail. Diffusion weighted imaging (DWI) allows the *in vivo* analysis of the microstructural changes which accompany normal and abnormal brain development, while functional MRI enables the impact of these changes upon functional connectivity to be examined (40). Newer modalities such as dynamic contrast enhanced MRI (41), MR elastography (42) and multiple echo time arterial spin labeling (43) may also herald a new horizon of neurodiagnostic evaluation.

Normal maturation in the perinatal period is correlated with a number of changes on DWI. These are thought to represent the loss of radial glia with an increase in myelination and dendritic arborisation during this period (44, 45). The apparent diffusion coefficient (ADC), a measure of the diffusion of water molecules within tissues, provides a sensitive measure for changes in tissue microstructure. ADC decreases in both white and gray matter during development as increasing complexity in these tissues restricts diffusivity (40, 46–48). Fractional anisotropy (FA) also measures water diffusion, but takes into account the directionality of this diffusion, providing insights into cellular organization and structure. It is particularly useful for examining white matter tracts. During the perinatal period FA increases in white matter but decreases in the gray matter (48, 49).

In those born prematurely, imaging abnormalities are characterized by cerebellar, cortical and subcortical volume loss, with associated abnormalities of thalamocortical connections (44, 47, 50). Abnormalities of thalamocortical connectivity are reiterated by resting state MRI studies, which can analyse the development of neuronal networks by detecting the fluctuations in blood flow to different brain areas. These studies have demonstrated resting state networks in preterm infants at term age to be underdeveloped when compared to term-born controls (51). These deficits persist to school age, where impaired performance in auditory language tasks is associated with alterations in neuronal connectivity in cortical language regions (52). The level of connectivity between the thalamus and a number of cortical regions by term age is correlated with cognitive scores at 2 years, while the change in FA on serial scans in those born prematurely has been suggested to be a predictor of long-term neurodevelopmental outcome (48, 50).

GMH-IVH is associated with widespread imaging abnormalities in addition to those associated with prematurity. These include abnormalities within central, parietal and occipital white matter tracts and cerebellar volume reduction (48, 53). Many of these abnormalities are present even after low grade GMH-IVH, (47, 48, 53, 54) while intraventricular blood loads too small to be detected by sonography are still associated with significant white matter damage (55). These microstructural abnormalities predict long term deficits in motor and cognitive functioning (53). However, the mechanism behind these abnormalities remain poorly understood, with little progress made in past decades to improve the considerable morbidity associated with GMH-IVH (56).

### Reducing CSF Toxicity May Improve Outcome

In an attempt to negate the deleterious impact of intraventricular blood, the DRIFT study (drainage, irrigation and fibrinolysis) was set up to establish if continuous ventricular irrigation over 72 h, to wash out bloodstained CSF and breakdown residual clot, could reduce the rate of progressive hydrocephalus and improve neurological outcome (57). Irrigation was achieved using 2 implanted ventricular catheters (right frontal & left occipital) and required intensive monitoring within a neonatal intensive care unit, with precise assessment of fluid inflow and outflow and continuous measurement of intracranial pressure.

The DRIFT study was discontinued due to concerns related to the safety of prolonged ventricular irrigation and the incidence of re-bleeding related to use of the thrombolytic agent tissue plasminogen activator (t-PA). Although the DRIFT study did not demonstrate a reduction in the requirement for a permanent VP shunt, cognitive and motor follow up at 2 and 10 years did demonstrate a significant reduction in the rate of death and severe disability in the DRIFT group compared to the group that received standard treatment (22, 25). Implicating that reducing CSF toxicity is a viable approach to treatment.

Building on the findings of the DRIFT trial, neuroendoscopic clot lavage at the time of temporizing intervention (e.g., ventricular subgaleal shunt or ventricular access device) has been proposed (58) (Figure 1). This approach reduces the risk associated with 72 h of irrigation to a single operative procedure and removes the need for prolonged drainage on the neonatal intensive care unit, also the mechanical effect of direct endoscopic lavage obviates the need for a fibrinolytic drug, which, in the DRIFT study was associated with a significant rebleeding rate. Early results have shown a potential reduction in the rate of hydrocephalus (59, 60) but more research is needed to understand the potential role of neuroendoscopic washout for the treatment of NIVH.

It is also important to consider that ventricular irrigation is undertaken on average between 20 and 30 days following hemorrhage. This is due to the intrinsic instability of the premature neonate and the high risk of intervening at earlier time points. In addition, intervention is currently only considered in neonates with progressive ventriculomegaly or signs and symptoms indicative of raised intraventricular pressure. As such, current therapeutic trials, focused on ventricular irrigation after the bleed, will have no impact on prior toxic damage. Nor will it be of any benefit to those neonates with isolated IVH i.e., without progression to post haemorrhagic hydrocephalus. By understanding the mechanisms through which early toxicity impacts on the periventricular tissue, early therapeutic strategies to limit brain injury and improve neurological outcome in babies with IVH can be developed.

### Early Intervention Before the Onset of Symptoms May Improve Outcome

The timing of temporizing intervention in the treatment of NIVH remains contentious with two schools of thought persisting. The first advocates for so-called ‘early intervention’, guided by the size of the ventricles as measured by transfontanelle cranial ultrasound scan (cUSS). An increase in the ventricular index (VI) beyond the 97th+4 mm centile (based on the Levene chart) would in the first instance trigger a lumbar puncture, if this fails to prevent ventricular enlargement a temporizing intervention would then be inserted [ventricular subgaleal shunt (VSGS) or ventricular access device (VAD)] (Figure 1) with regular CSF aspiration to maintain the ventricular size below the 97th +4 mm centile. If repeated aspirations are subsequently required to maintain the VI below the 97th +4 mm centile then once the child was more than 2 kg in weight a permanent ventricular peritoneal shunt would be inserted. In contrast, the so-called “late intervention” group advocate for temporizing intervention only if the child demonstrates signs or symptoms indicative of raised intraventricular pressure, for example a rapid increase in the head circumference (more than 2 mm per day) a tense anterior fontanelle, splaying of the cranial sutures, new eye signs such as an up-gaze palsy or sunsetting, or cardiovascular instability such as apneic or bradycardic events thought to be attributable to raised intraventricular pressure.

The weight of evidence would seem to insinuate that early intervention before the onset of symptoms improves outcome (61), with two randomized controlled trials now supporting this position (22, 25, 62). Similarly, a retrospective review by Leijser et al. comparing outcome in the early vs. late groups (26) showed that neurodevelopmental outcome at 2 years was significantly improved in the early intervention group i.e., intervention based on an increase in ventricular size rather than the onset of symptoms. This position was further supported in a meta-analysis comparing how the timing of temporizing intervention impacted on outcome (63). However, this enthusiasm has to be tempered by the potential risk posed by surgical intervention in this extremely vulnerable patient population.

For the purposes of this review, it is plausible that prolonged exposure to blood-stained CSF, under higher than physiological pressure, increases the potential for harm to the ciliated ependyma. This in turn may increase the degree of ependymal denudation, as such I postulate that earlier intervention i.e., prior to the onset of symptoms, has the effect of preserving the ciliated ependymal border and this, in part, may contribute to the improved outcome seen in the early intervention groups.

## The Role of the CSF Microenvironment in Maintaining Normal Brain Development

**Figure 3:**
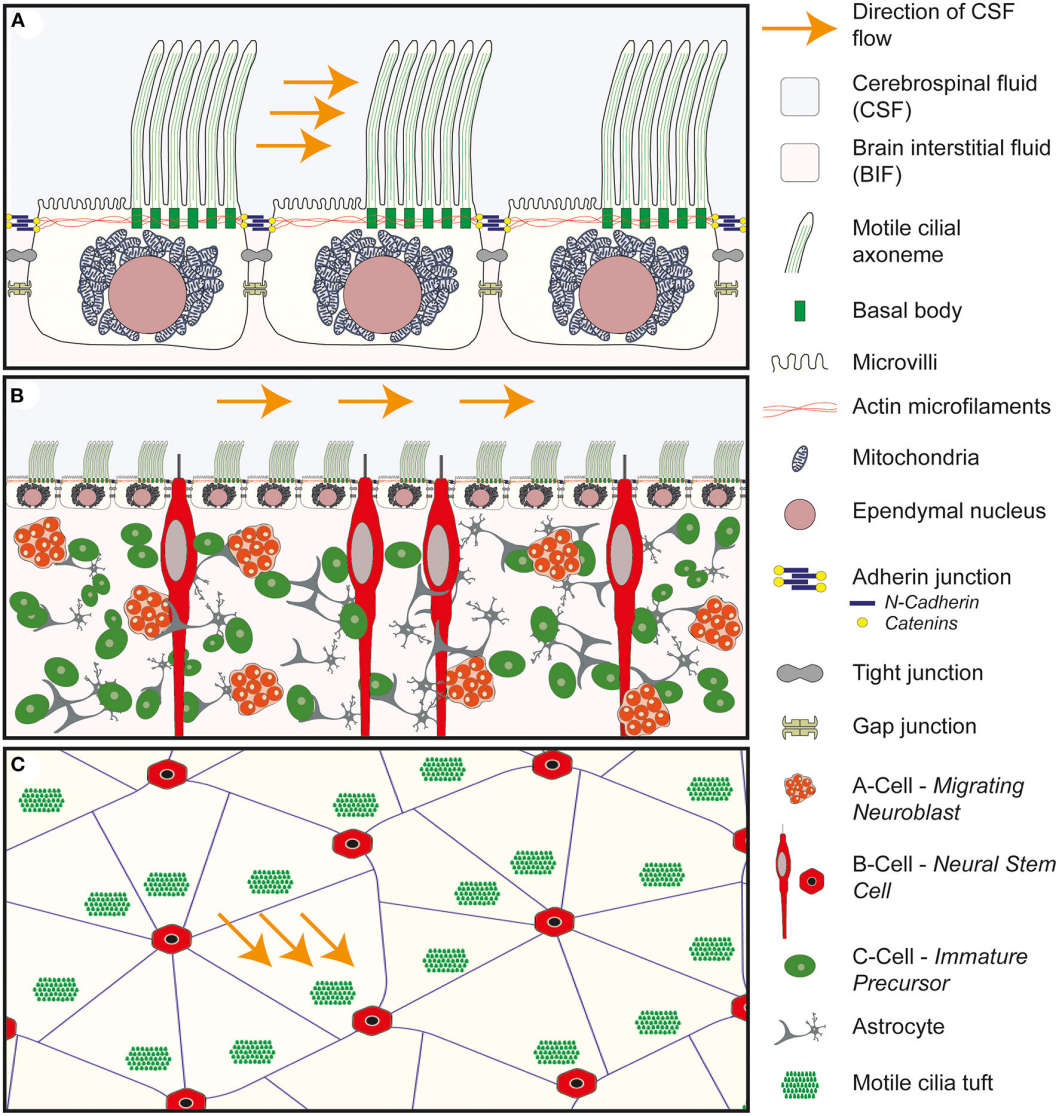
Anatomy of the wall of the lateral ventricle. **(A)** Anatomy of the E1 ependymal cilia. Multiple motile cilia are shown arising from basal bodies within the apical/ventricular surface of the cell. The basal bodies are shown to be tightly integrated with the actin microfilaments which maintain the structure of the ependymal cell. The motile cilia are asymmetrically distributed across the surface of the cell and found in a tuft of cilia in a downstream position in relation to the flow of CSF. The remainder of the surface is covered in microvilli. Deep to the apical surface the large spherical shaped nucleus is seen surrounded by mitochondria. Connecting the ependymal cells together are three cell to cell junctions: the adherin junction, consisting of n-cadherin and catenins, tight junctions and gap junctions. **(B)** Cross-sectional anatomy of the wall of the lateral ventricle demonstrating the relationship between the ependymal layer and the underlying neural stem progenitor cells within the subventricular zone. The ependymal layer sits at the interface of the cerebrospinal fluid (CSF) and the brain interstitial fluid (BIF) and acts as a gatekeeper, modulating the brain microenvironment surrounding the NSPC. **(C)** Enface anatomy of the wall of the lateral ventricle. The tuft of motile cilia are seen to arise from a downstream position in relation to the flow of CSF. The E1 ependymal cells are arranged in a pin-wheel orientation around the cilia of the b-cell.

Throughout embryological and fetal development, the neuraxis is expanded and populated by cells derived from the ventricular and subventricular zones of the developing brain (64) (Figure 3B). Cell intrinsic and microenvironmental cues combine to coordinate precise temporal and spatial proliferation and differentiation of the neural stem progenitor cells (NSPC) within the subventricular zone (65), resulting in remarkable regional specificity and the formation of the incredible diversity and complexity of cells seen within the brain and spinal cord. This process has been shown to continue through the third trimester into the early postnatal period (66, 67).

The microenvironment surrounding the NSPC within the subventricular zone of the periventricular region, is in part reliant on the composition of the CSF generated by the choroid plexus and periventricular epithelial cells (68) and the degree of interchange between the brain interstitial fluid and the cerebrospinal fluid (69, 70). In particular the CSF is thought to contribute a neurohumoral route influencing brain development (71–73).

Evidence to support the neurohumoral gradient within the CSF comes from the differences seen between the ventricular and lumbar CSF (74), the region specific transcriptomes of the choroid plexus in different regions of the mouse embryo (75) and the detection of hormones and neuropeptides secreted into the CSF (71). Approximately 80% of CSF proteins are thought to arise from the serum through a process of filtration at the blood brain barrier within the choroid plexus. The remaining 20% arising from the brain parenchyma; neurons, glia and secretory cells lining the walls of the ventricle (74, 76). These excreted glycoproteins within the CSF set up a specific neurohormonal gradient assisting in the precise temporal and spatial control of NSPC division within the SVZ. A particular role for the subcommissural organ, which sits at the opening of the third ventricle into the cerebral aqueduct and is the source of Reisner's fiber has been proposed to play a role in patterning within the spinal cord (72).

In addition to the modulation of the microenvironment within the SVZ, the neural stem cells (NSC) (also known as B cells) arising from the germinal matrix within the subventricular zone extend primary cilia through the ependymal lining of the ventricle (77, 78) and respond to neurohumoral developmental cues arising from the CSF (71) (Figure 3B). For example, intraventricular infusions of Fibroblast Growth Factor (FGF) & Epidermal Growth Factor (EGF) have been shown to increase proliferation within the SVZ. Similarly, other soluble factors within the CSF, including: Insulin like growth factor 2 (IGF2); Bone morphogenetic proteins (BMPs); Wnts; Sonic Hedgehog & Retinoic acid, have also been shown to influence NSPC behavior in the VZ/SVZ (79).

As such I postulate that changes in the proximal/apical domain (78) caused by IVH (changes in the CSF physiology) will impact on cortical development due to changes in the CSF microenvironment and disruption to ependymal integrity, and further to this may lead to disordered brain development contributing to the Neurodevelopmental disability seen following GMH/IVH. Understanding this process may give us an opportunity to introduce new treatment options.

### Changes to the Cerebrospinal Fluid as a Consequence of Intraventricular Hemorrhage

In addition to disturbing the delicate neurohumoral gradients with the CSF (as described above), NIVH also causes profound and toxic changes to the CSF microenvironment. The parenchymal origin of the hemorrhage classically lies within the germinal matrix around the head of the caudate nucleus. As the bleed ruptures through the ependymal wall of the ventricle it releases whole blood into the CSF. As a result, erythrocytes containing hemoglobin, plasma components, products of the coagulation cascade, platelets and leukocytes are all released into the CSF and all have been implicated in the pathogenesis of injury following intraventricular hemorrhage (80).

Haemolysis of the erythrocytes within the ventricular CSF leads to the release of hemoglobin which is further broken down through the activity of haem oxygenase resulting in the release of iron into the CSF, which has been shown to be raised in the CSF following neonatal IVH (81). The release of free iron into the CSF leads to the formation of free radicals and oxidative substances and there is considerable evidence that this process may contribute to the injury phenotype following IVH. For example, animal studies have shown that intraventricular injection of iron causes ventriculomegaly and brain damage, a phenotype reversed through iron chelation either with minocycline or deferoxamine. An alternative approach to iron chelation, exploiting the role of haptoglobin as a hemoglobin scavenger has also been proposed (82).

In addition to the erythrocytes, the release of TGFβ from the platelets has been shown and the presence of leucocytes in the CSF causes a short-lived local tissue reaction. In this regard, it is intriguing to note the association between chorioamnionitis and the increased risk of developing of posthaemorrhagic hydrocephalus (PHH), it is plausible that this pathological process is exacerbated by the release of inflammatory mediators from the serum into the CSF causing an inflammatory reaction.

In addition to the toxic metabolites excreted from the blood components within the CSF, exposure of the ependyma and underlying parenchyma to this toxic CSF milieu causes inflammation and local reaction within the periventricular tissues, resulting in the migration and activation of microglia (9). Analysis of cytokine and chemokine levels from the lumbar CSF of neonates following intraventricular hemorrhage, revealed that among 17 CSF biomarkers, 8 were significantly increased (IL-1α, IL-4, IL-6, IL-12, TNF-α, CCL-3, CCL-19, and CXCL-10) and one was significantly decreased (XCL-1) in IVH in comparison to control (83). Similarly, CSF osmolarity was found to be higher in PHH with increased levels Na^+^ K^+^ & Cl^−^ and decreased glucose (84).

As time progresses the acute inflammatory response is thought to be superseded by a chronic inflammatory condition (85, 86), which has been postulated to be causative for maintaining the hydrocephalic state, resulting in a specific CSF phenotype in neonates with posthaemorrhagic hydrocephalus (87).

## The Anatomy of the Wall of the Ventricular System

In 1836 Purkinje wrote an essay on the Ueber Flimmerbewegungen im Gehirn, which translates as “About flickering movements in the brain” describing the activity of ependymal cells in the wall of the sheep lateral ventricle. The ependymal cell lining of a fluid filled ventricular system within the brain is highly conserved throughout phylogeny. Whilst the physiological role of the CSF and its interaction with the ependymal cell lining of the ventricular system remains to be definitively determined, a potential role for modulating the brain microenvironment has been proposed. Multiple different cells types with region specific anatomy and complex branching axonal systems linking cells have been identified. Similarly the cilial beat frequency and pattern have been shown to be highly coordinated and responsive to changes in CSF composition further implicating a physiological role in maintaining homeostasis.

The wall of the ventricular system is lined almost entirely by multiciliated E1 ependymal cells. The exceptions being the area postrema at the caudal end of the fourth ventricle and the subcommisural organ at the transitional zone between the roof of the third ventricle and the cerebral aqueduct (88). This single cell layer sits at the interface of the brain parenchyma on its basal surface and the cerebrospinal fluid at its apical/ventricular surface. This highly specialized and continuous lining of the ventricle is easily recognizable using simple staining and light microscopy due to the cuboidal shape of the E1 cell (Figure 3A) and the regimented alignment of the large spherical nuclei lining the border of the lateral ventricle (Figure 3B).

Projecting out from the apical/ventricular border of the E1 cell is a tuft of motile cilia which beat in a coordinated and rhythmical fashion contributing to the propulsion of CSF at the ventricular surface, a process referred to as laminar flow of CSF. The E1 cells are held tightly together with adherin, gap and tight junctions which contribute to a strong functional barrier between the CSF and the brain parenchyma. By modulating the passage of water and ions across the apical and basal borders, the E1 cells have been postulated to act as gatekeepers, controlling influx and efflux across the CSF/brain interstitial fluid (BIF) interface (71). As such the integrity of the ependymal border is thought to afford a degree of protection to the underlying brain parenchyma and to play a role in maintaining brain homeostasis.

Interspersed between the E1 cells, other highly specialized cell types are also recognized. These differ from the E1 cells in that rather than multiple cilia (multiciliated), these cells exhibit single (monociliated) or paired (biciliated) cilia and in contrast to the motile cilia seen on the E1 cell, these cilia are non-motile or so-called primary cilia. These non-motile cilia are thought to play a sensory role, acting as biosensors, detecting changes in mechanical and chemical stimuli within the CSF (89, 90). In addition they may play a role in absorbing substances from (90) and secreting substances into the CSF (76).

The wall of the ventricular system is therefore though to play a significant role in maintaining brain homeostasis, both through its protective function, as a continuous border between the CSF and the parenchyma, and as a gatekeeper, controlling influx and efflux between the CSF and the brain interstitial fluid (BIF), but also in a more nuanced fashion sensing and modulating the composition of the CSF and relaying this information to distant targets within the brain.

### Development of the Wall of the Lateral Ventricle From the Neuroepithelial Lining of the Neural Tube

During embryological development, the neural tube is lined with neuroepithelial cells which, through a combination of cell intrinsic and microenvironmental cues, symmetrically and asymmetrically divide to produce further stem cells and progenitor cells which populate the parenchyma. Late in the second trimester (91) around the 25th week of gestation (92) a subpopulation of the neuroepithelial cells, in contact with the primitive CSF, undergo a conformational change and transform to generate the ciliated ependyma (93). A process which is thought to occur in a caudal to rostral direction in humans (91).

This switch from neuroepithelial progenitor cell to multiciliated ependymal cell necessitates that the neuroepithelial cell which contains one single centrosome and associated primary cilia, is transformed into a polarized cell with tens of centriolar structures nucleating a functional motile ciliary tuft (94). Whilst this process has been extensively studied, with excellent reviews available in the literature (94, 95), the molecular mechanisms controlling this transformation remain to be definitively elucidated.

In brief this process is thought to be reliant on Notch inhibition which results in an upregulation of multicilin, a protein required for the formation of multiciliated cells (94). Three essential stages have been described in the formation of the ciliated ependyma; the amplification stage, in which massive amplification of centriole production occurs, the migration phase in which the newly formed centrosomes separate off and migrate to the apical surface to become the basal bodies of the ependymal cell and finally the production of cilia arising from the newly formed basal bodies which project into the ventricular lumen. Each of these steps can malfunction resulting in faulty cilia (94).

### The Microscopic and Functional Anatomy of the E1 Ependymal Cell and Its Motile Cilia

Multiciliated E1 Cells are CD24, S100β, FoxJ1, Sox2 and CD133 positive, and Nestin & GFAP negative. They are cuboidal in shape with a large spherical nucleus surrounded by multiple mitochondria. The lateral processes are interdigitated and held together by apical adherin junctions, tight junctional complexes and gap junctions (96) (Figure 3A).

In contrast to other multiciliated cells in the body (97), which exhibit tufts of cilia evenly distributed across the apical surface (for example in the respiratory or urogenital tracts), the cilia of the E1 cell are confined to a specific region of the apical surface which lies in a downstream position (in relation to the direction of CSF flow), a property referred to as translational polarity (Figures 3A–C). The so called basal body patch from which the cilia arise constitutes around 4–35% of the ventricular surface of the ependymal cell (96) and the primary cilium of the neuroepithelial progenitor cell has been shown to play an important role in coordinating this property of translational polarity (98). The remaining apical surface of the E1 cell is covered in microvilli which significantly increase the apical surface area of the cell but their physiological role remains unclear.

The individual cilia also exhibit rotational polarity, this refers to the fact that the cilia all align in the same orientation within the cilial tuft, such that they beat in the same direction to ensure coordination in the direction of travel of CSF flow.

The cilia themselves are microtubule based hair-like organelles (99) which project into the ventricular lumen. They arise from a basal body within the apical membrane and exhibit a complex anatomy which changes from its origin at the basal body to the tip of the cilia (99). The main body of the cilia is composed of nine pairs of microtubules, arranged in a cylindrical pattern, surrounding an extra central pair, which is referred to as the 9 + 2 arrangement (93). The outer ring of microtubules is connected to the central pair via inner and outer dynein arms. These axonemal dyneins create sliding interactions between the microtubules (100) facilitating a ratcheting movement and allowing the cilia to bend. The cilia have been shown to exhibit an effective stroke in which the cilia bend at the base propelling the CSF forward and a recovery stroke in which the cilia readopts its original position.

The 9 + 2 arrangement is referred to as the ciliary axoneme and is evolutionarily well-maintained throughout phylogeny (100). Up to 50 motile cilia measuring between 8 and 15 μm arise from the ependymal surface (96). The cilia beat in a highly coordinated metachronal wave, synchronized along the longitudinal axis. This is postulated to be due to tight coupling of the E1 cells through gap junctions.

Interestingly it has been shown that the ependymal cilial beat frequency (CBF) may be responsive to changes in the CSF microenvironment, for example the metabolic peptide melanin concentrating hormone (MCH), has been shown to regulate cilial beat frequency (101). Similarly, the neurotransmitter serotonin has been shown to increase the beat frequency (96, 102) in a calcium dependent manner, whilst adenosine receptor agonists and ATP have been shown to reduce CBF, through activation of the A_2B_ receptor (103) and through a calcium independent cAMP mediated pathway (102), respectively. The physiological role of this mechanism remains to be determined.

### Other Ciliated Cells Lining the Walls of the Ventricular System

As discussed above, around 90–95% of the surface of the ventricle is covered by the E1 ependymal cells but interspersed between the tufts of motile cilia are non-motile cilia arising from different specialized cell types (Figure 3C). Monociliated B-cells or neural stem cells residing within the subventricular zone are seen to extend a process between the E1 cells, similarly tanycytes a specialized type of monociliated ependymal cell are seen, predominantly around the wall of the third ventricle. In addition biciliated E2 ependymal cells have also been described. In contrast to the active control of CSF laminar flow, these non-motile cilia are thought to function as biosensors detecting mechanical and chemical stimuli (89).

#### B-Cells – Neural Stem Progenitor Cells of the Sub-ventricular Zone

As discussed above, the ependymal cells lining the ventricular system arise embryologically at around 25 weeks gestation from terminal differentiation of the neuroepithelial cells of the primitive neural tube. Lying on the basal surface, deep to the ependymal layer, is the subventricular zone, this layer is recognized as a germinal zone containing neural stem progenitor cells of the developing brain.

In the mouse, whole mount staining of the wall of the lateral ventricle with β-catenin, to show the apical adherin junctions between the ependymal cells, and γ-tubulin to show the position of the cilia, demonstrates an elegant pinwheel structure with the primary cilium of the B-cell surrounded by the multiple motile cilia of the ependymal cells (77) (Figure 3C). This arrangement has also been described transiently in the developing human ependymal wall (91). It is interesting to note that the processes of the B-cells which pierce through the ependymal lining to access the CSF, are also tightly bound to surrounding ependymal cells by adherin junctions (91) further reinforcing the potential barrier function that the ependyma plays.

Activity of the B-cells has been shown to be influenced through changes in the CSF microenvironment, for example fibroblast growth factor (FGF) & epidermal growth factor (EGF) infusions have been shown to increase proliferation, whilst other soluble factors within the CSF, including: Insulin like growth factor 2-IGF2; Bone morphogenetic proteins (BMPs); Wnts; sonic hedgehog (SHH) & retinoic acid, have also been shown to influence neural stem progenitor cell (NSPC) behavior in the subventricular zone (SVZ) (79), a process which is thought to be modulated by the B1 cell cilium.

#### Tanycytes–Contacting Distant Targets Within the Brain

Tanycytes are bipolar mono-ciliated cells with microvilli on the apical/ventricular surface. They are found predominantly in the walls of third ventricle although a few have been observed in the lateral walls of the lateral ventricles (104) and in discrete locations around the central canal of the spinal cord (95). The primary non-motile cilia of the tanycyte (9+0) has been shown to exhibit functional receptors for GABA and glutamate (96) and are postulated to link changes in the CSF microenvironment to neuroendocrine events (101).

The long basal process of the tanycytes arising from the wall of the third ventricle connect with the thalamus and hypothalamus and are thought to play a role in regulating their function (105). It has been postulated that the high prevalence of obesity in ciliopathic syndromes, such as Bardet Biedl Syndrome (BBS) and Alstrom Syndrome may be related to impaired CSF to hypothalamic signaling, potentially implicating a role for the cilia and specifically the tanycytes in controlling food intake (73) and modulating satiety. Tanycytes have also been shown to communicate with the portal blood system and may play a secretory role (106) alternatively the tanycytes may transport compounds from the CSF to their terminals (90).

## Ependymal Denudation/Ventricular Zone Disruption Secondary to Intraventricular Hemorrhage

The term denudation has been used to describe the stripping away of the ependymal lining from the wall of the lateral ventricle. It was first coined by Jiménez et al. (107) in reference to the phenomenon of ependymal layer damage seen embryologically in the Hyh mouse, a genetic strain of mouse recognized to develop spontaneous hydrocephalus. The association between hydrocephalus and pathological changes in the ependyma have been recognized and reported for many years (108). The changes seen to the ependyma lining the wall of the ventricle were thought to result as a consequence of the sheer and stress forces associated with dilation of the ventricle i.e., as a result of or secondary to the development of hydrocephalus (88). Work on the Hyh mouse implicated that denudation of the ependyma preceded the development of hydrocephalus and was therefore thought to be causative in the development of hydrocephalus (see section below).

Unlike other ciliated epithelial surfaces, the ciliated ependymal lining of the brain ventricular surface undergoes minimal repair or renewal in response to aging or injury (94) in this regard, autopsy studies in adults show that small patches of ependymal loss are commonly seen. Some have taken this as an indication that the role of the ependyma is reduced through life and whilst the function of the ependyma may be critical in early development (as described above) it may not play an ongoing significant role through life. Conversely, others have argued that ependymal denudation is not normal at any age (88) and further to this have associated areas of ependymal loss in the adult with damage to the underlying parenchyma, specifically correlating areas of gliosis deep to sections of ependymal loss, and have postulated a link between ependymal health and neurodegenerative (109) and neuropsychiatric disease (110).

### Disordered Cell Division Within the SVZ Deep to Areas of Ependymal Denudation

The site of hemorrhage in NIVH commonly arises within the germinal matrix around the head of the caudate nucleus. Ki67 staining in the immediate perihaematoma region has demonstrated reduced activity of the NSPC (111) implicating a direct toxic effect of blood on the activity of the NSPC within the SVZ. *In vivo* animal studies modeling NIVH (112–114) and *in vitro* analysis of the effect of blood products on cell culture (115) have also demonstrated an adverse impact on the Neural Stem Cell niche.

Away from the site of the hemorrhage the secondary impact of NIVH appears more nuanced. Areas of damage and disordered activation of the NSPC within the SVZ (114, 116) are seen underlying patches of ependymal denudation. The mechanism underlying ependymal denudation remains incompletely understood but detailed autopsy studies in hydrocephalus (117) spina bifida (118) and following neonatal intraventricular hemorrhage (119) have implicated that denudation may be related to changes in cell adhesion between the ependymal cells, for example, prior to denudation, cytoplasmic translocation of the junctional protein N-Cadherin has been described (118, 119).

**Figure 4:**
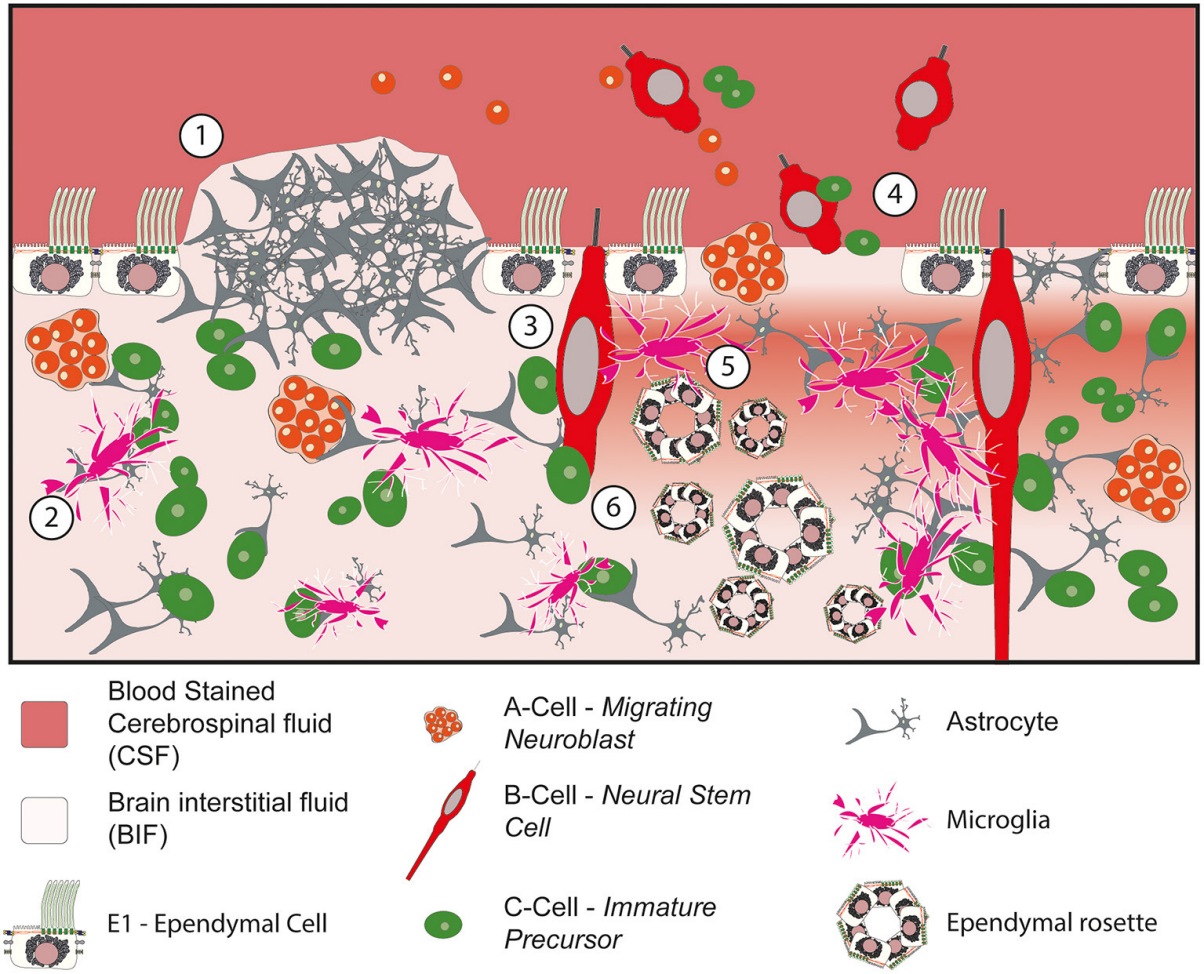
Impact of denudation of the ependyma within the ventricular and subventricular zones. (1) Subventricular glial nodule bulging into the ventricular lumen. The ependymal surface is discontinuous over the surface of the nodule but islands of ependymal cells remain intact in the surrounding vicinity. (2) Migration and activation of microglia into the subventricular zone. (3) Damage to the neural stem progenitor cells with exposure to bloodstained CSF. (4) Loss of ependymal cells within the lining of the ventricle causes cells to escape into the CSF. (5) Loss of homeostasis at the CSF to BIF border causes periventricular egress of CSF, seen as a band of oedema/hypodensity in the subventricular zone. (6) Ependymal rosettes are seen deep to areas of ependymal denudation within the subventricular zone.

Ultimately, the loss of the ciliated ependymal border has been shown to cause a constellation of different abnormalities within the ventricular zone (Figure 4). For example, the loss of the protective ependymal layer results in the eruption of cells, including neural progenitor cells, into the ventricle (120). Periventricular heterotopia and ependymal rosettes are also seen. In addition, damage to the ependymal border may contribute to or exacerbate the ingress of CSF into the subventricular tissue resulting in a subventricular band of increased water density designated as transependymal oedema (88). Following this acute phase, microglial infiltration into the SVZ associated with the formation of extensive gliotic nodules below the surface of the ependyma occurs (117–119). This gliotic patch may serve to prevent further loss of cells into the ventricle in an attempt to restore homeostasis.

In addition to the role of the ependyma in protecting the underlying subventricular zone and acting as the gatekeeper in regulating the periventricular brain microenvironment, the integrity of the ependymal surface is thought to directly influence normal cell division and proliferation within the subventricular zone. For example, the intercellular junctions between ependymal cells and the NSPC have been shown to play a critical role in the control of neurogenesis. The junctional protein ankyrin plays an important role is stabilizing the ependymal cell and loss of this protein (through FoxJ1 knockout) causes a reduction in neurogenesis (121). Similarly, ependymal cells produce Noggin, which promotes progenitor proliferation and neuroblast formation both *in vitro* and *in vivo*, implicating that the ependyma is able to directly modulate the activity of the NSPC (78).

In addition to the loss of the E1 ependymal cell, IVH also potentially impacts on the primary non-motile cilia of the B-cells, E2 cells and tanycytes. Several G protein coupled receptors have been identified in primary cilium [for example sonic hedgehog (68)] which are known to specialize the neuronal cell type in the developing neural tube. Further to this it was recently shown that activation of epithelial sodium channels in the base of the B-cell primary cilia, secondary to fluid movement, regulates the proliferation of the neural stem cells (122, 123) implicating a mechanosensory function of the CSF to cilial interaction. As such in addition to being sensitive to changes in the components of the CSF, fluid movement and directionality also influence cell division and consequently brain development (65).

### Disordered Cell Migration Following IVH

In addition to direct injury to the NSPC in areas of ependymal denudation (as discussed above), it has been shown that damage to the motile cilia may impact on cell migration out of the SVZ (124). Using whole mount sections from the wall of the lateral ventricle Sawamoto et al. (124) showed that damage to the motile cilia resulted in disordered cell migration out of the SVZ. Further evidence to support the role of the motile ependymal cilia in guiding the migration of the neural precursor cells comes from genetic studies of periventricular heterotopia. Cell migration out of the SVZ was known to be impaired in FLNA and ARFGEF2 transgenic mouse models, this was initially thought to be due to an intrinsic failure of cell migration, but detailed analysis showed that early born (FOXP1) neurons were able to populate the cortex normally and it is the late born (CUX) neurons which fail to integrate. Examination of the ependyma demonstrates a loss of neuroependymal integrity (125) which is implicated as the cause of periventricular heterotopia and failure of cell migration in these genetic models.

The majority of cells generated by the SVZ in the third trimester and early postnatal period are thought to be interneurons (91) with three specific migratory routes out of the SVZ described: (1) the arc pathway distributing interneurons to the frontal lobe; (2) the medial migratory stream (MMS) to the medial prefrontal cortex; (3) the rostral migratory stream (RMS) to the olfactory bulb (66, 67).

### The Association Between Cilial Activity and Hydrocephalus

Historically it was believed that blood products within the CSF caused a communicating hydrocephalus due to a blockage of CSF reabsorption at the arachnoid granulations. Whilst this may play a contributory role, the fact that arachnoid granulations are not developed in the neonate implies that other mechanisms may also be responsible.

Fundamental shifts in our understanding of the etiology of hydrocephalus, CSF physiology and the role of CSF movement through the brain parenchyma and neuraxis (69, 70) have thrown into question the contribution that ependymal cilia play in the pathogenesis of hydrocephalus. It is evident that damage or denudation of the ependyma will impact on the laminar flow of CSF at the wall of the lateral ventricle, a mechanism which may be particularly relevant at “pinch points” within the ventricular system, including the foramens of Monroe, Magendie and Lushka, and through the cerebral aqueduct (117, 126). For example, ependymal cell loss secondary to viral infection has been shown to cause ependymal cell shedding within the cerebral aqueduct a mechanism which is thought to play a role in the development of hydrocephalus following fetal infection (126). However, transgenic models have shown that manipulation of genes important in ependymal function cause hydrocephalus both through an impact on the cerebral aqueduct and also hydrocephalus without an impact on the cerebral aqueduct (127) implicating that loss of laminar flow may not be the only mechanism through which ependymal denudation causes hydrocephalus. It is also important to note that whilst transgenic models of primary ciliary dyskinesia (PCD) commonly develop hydrocephalus secondary to altered ependymal cilial function (128), and the targeted ablation of Kif3 genes, which are responsible for ciliogenesis, show severe congenital hydrocephalus in utero (129), the rates of hydrocephalus seen in humans with PCD is significantly lower (130).

Other potential mechanisms linking ependymal damage to the pathogenesis of hydrocephalus include disruption of a proposed feedback loop between the cilia of the ependyma and choroid plexus and consequently on CSF production. For example Banizs et al. demonstrated that in the Tg737orpk mouse, which has defects in cilia assembly secondary to a mutation in the Polaris gene, hydrocephalus was present before the motile cilia were formed (131). Further they showed that dysfunctional motile cilia impact on the cAMP within the choroid plexus. Similarly, Narita et al. (89) proposed that the cilia of the choroid plexus exerted a tonic inhibition of CSF production through activation of the neuropeptide FF (NPFF) receptor and showed in cell culture that stripping the cilia of the choroid plexus cells resulted in increased fluid transcytosis.

Exposure of the ependyma and choroid to blood products results in changes in aquaporin RNA expression and protein expression (80, 132) a phenomenon also seen in areas of ependymal denudation (109), with a redistribution of aquaporin expression from perivascular (within 15μm of the vessel) to stromal (>15 μm from the vessel) reported (133). Aquaporins undoubtedly play a role in water transport within the brain but their exact role in the pathogenesis of hydrocephalus remains to be fully determined.

Damage to the ependyma results in astrogliosis within the periventricular tissues (134) and the repair of areas of ependymal loss with subventricular glial nodules which bulge into the ventricular lumen (88) (Figure 4). Over time this scarring may impact on the degree of elasticity within the wall of the lateral ventricle. This loss of compliance within the wall of the lateral ventricle has been directly associated the development of hydrocephalus, indeed a recent study demonstrated that inhibiting periventricular astrogliosis significantly reduced the incidence of hydrocephalus (133). Loss of compliance may also impact on glymphatic transport of CSF through the brain parenchyma. The glymphatic system is thought to be dependent on the transmission of arterial pulsations to propel CSF through the parenchyma (43) and given that the glymphatic system and cribriform lymphatics may play a disproportionately important and leading role in CSF absorption in the neonate, the loss of compliance in the wall of the lateral ventricle may be relevant.

Finally, an important and evolving concept is the finding of inflammation induced choroid plexus hypersecretion secondary to intraventricular hemorrhage. Using a mouse IVH model, Karimy et al. (86) demonstrated that blood within the ventricle, in this case methaemoglobin and iron, were sufficient to cause activation of the toll like receptor (TLR4) in the apical border of the choroid plexus epithelium. This was shown to cause a nuclear translocation of NF-κB, and ultimately to increase CSF production, in part through increased phosphorylation of the NKCC1 transporter in the apical membrane of the choroid plexus epithelium (85).

## Summary

Despite concerted efforts to reduce the incidence of premature birth and the incidence and severity of neonatal intraventricular hemorrhage, it remains a fact that NIVH is still a frequent problem encountered by neonatologists and pediatric neurosurgeons alike. Given the extreme vulnerability and fragility of this patient group, the optimal treatment regime is not clear and NIVH remains the most common cause of hydrocephalus in the pediatric population (post haemorrhagic hydrocephalus) and a significant cause of neurodisability in children.

Brain development in the final trimester and early postnatal period is reliant on both the maintenance of a neurohumoral gradient within the CSF (114) and the integrity of cilial signaling across the ependymal border (124). Neonatal intraventricular hemorrhage impacts on both of these processes at a critical time in brain development (64).

Traditional treatment paradigms based on a temporizing surgical intervention, with CSF diversion only in cases of symptomatic ventriculomegaly/raised intraventricular pressure have recently been challenged by new evidence showing that washing away bloodstained CSF (21, 22) and also earlier intervention prior to the development of clinical symptoms (26), may improve outcome.

These are undoubtedly important and thought-provoking developments in the field, but by virtue of the fact that these neonates are extremely fragile and ventriculomegaly takes time to evolve, surgical intervention will not normally be considered until around 20–30 days of life. As such, this paradigm of surgical intervention, does not address the potential early toxicity caused due to the release of blood into the CSF.

In order to develop new therapies to combat the early toxicity of NIVH more research is needed to understand both the changes that occur in the CSF secondary to hemorrhage and further to this, the impact that these changes have on the developing brain. Specifically, we need to increase our understanding of the mechanisms underlying ependymal denudation and the consequences that this process has on the underlying periventricular cells within the subventricular zone.

The concept of earlier intervention prior to the development of symptoms (26) i.e., intervening in a clinically well-child, is challenging. It necessitates the field moving from a position of treating raised intraventricular pressure to instead instigating therapy to promote white matter survival and protection. Progressive ventriculomegaly, even in the absence of overt clinical signs and symptoms, should be interpreted as an indication that the CSF physiology is no longer in harmony, a process which is harmful to the developing brain and in particular the developing periventricular white matter.

Developing new therapies to treat the CSF with the intention of; reducing its toxicity, re-establishing haemostasis, and protecting the functional integrity of the ependyma, may herald new horizons in treatment. Given the difficulties associated with early surgical intervention, it may be that a staged treatment protocol would be most beneficial. For example, early medical treatment with the intention of buying time, whilst mitigating damage to the ciliated ependymal lining of the ventricle, followed by early instigation of endoscopic washout, timed to optimize the chance of facilitating clot and bloodstained CSF removal, whilst limiting potential risk to the neonate.

## Author Contributions

WD is responsible for the conception, curation, writing of this manuscript, and production of the figures.

## Funding

This work was supported by Pump Priming Grants from the Royal College of Surgeons and is supported by the NIHR GOSH BRC.

## Author Disclaimer

The views expressed are those of the author(s) and not necessarily those of the NHS, the NIHR or the Department of Health.

## Conflict of Interest

The author declares that the research was conducted in the absence of any commercial or financial relationships that could be construed as a potential conflict of interest.

## Publisher's Note

All claims expressed in this article are solely those of the authors and do not necessarily represent those of their affiliated organizations, or those of the publisher, the editors and the reviewers. Any product that may be evaluated in this article, or claim that may be made by its manufacturer, is not guaranteed or endorsed by the publisher.
